# Complications of tracheostomy in children: a systematic review

**DOI:** 10.1016/j.bjorl.2020.12.006

**Published:** 2020-12-30

**Authors:** José Faibes Lubianca Neto, Octavia Carvalhal Castagno, Artur Koerig Schuster

**Affiliations:** a Universidade Federal de Ciências da Saúde de Porto Alegre (UFCSPA), Disciplina de Otorrinolaringologia (ORL) e Programa de Pós-Graduação em Pediatria, Porto Alegre, RS, Brazil; b Hospital da Criança Santo Antônio, Serviço de ORL Pediátrica, Programa Programa de Fellowship em ORL Pediátrica Otorrinolaringologia Pediátrica, Porto Alegre, RS, Brazil; c Santa Casa de Misericórdia de Porto Alegre (UFCSPA), Serviço de ORL, Programa de Residência Médica em Otorrinolaringologia, Porto Alegre, RS, Brazil

**Keywords:** Tracheostomy, Child, Complication, Mortality

## Abstract

**Introduction:**

Tracheostomy is a procedure that can be associated with several well-described complications in the literature, which can be divided into transoperative, early postoperative and late postoperative. When performed in children, these risks are more common than in adults.

**Objective:**

To perform a systematic review of complications, including deaths, in tracheostomized pediatric patients.

**Methods:**

A search was carried out for articles in the Latin American and Caribbean Health Sciences Literature and PubMed databases. Cohort studies and series reports were selected, in addition to systematic reviews, published between January 1978 and June 2020, with patients up to 18 years old, and written in English, Spanish or Portuguese.

**Results:**

1560 articles were found, of which 49 were included in this review. The average complication rate was 40%, which showed an association with age, birth weight, prematurity, comorbidities, and emergency procedures. The most common complications were cutaneous lesions and granulomas. Mortality related to the procedure reached up to 6% in children and was mainly related to cannula obstruction or accidental decannulation.

**Conclusion:**

Pediatric tracheostomy is associated with several complications. The tracheostomy-related mortality rate is low, but the overall mortality of tracheostomized patients is not negligible.

## Introduction

Tracheostomy is the term that describes the presence of an opening in the trachea, while tracheotomy refers only to the act of making an incision in the trachea. For a long time, its only indication was infectious and tumor diseases that cause upper airway obstruction.^1^ From the 20th century onward, the indications for tracheotomy increased. Currently, almost two thirds of tracheostomies are performed on children under 1 year of age and usually remain for longer periods, when compared to adults. The main indications in children are laryngotracheal stenoses, prolonged mechanical ventilation and the need for pulmonary hygiene maintenance.^2^

Several techniques have been described. The main differences are usually regarding the direction of the skin incision, the incision shape in the trachea and the suture or not of the trachea in the skin, also known as “stoma maturation”. Possible surgical complications can be divided into transoperative and postoperative, which are subdivided into early and late postoperative complications. The prevalence and distribution of such complications is highly variable in the literature. This study aims to determine the possible complications of tracheostomy in children, including the mortality rate associated with the procedure, dividing them according to their temporal relationship with the time of surgery.

## Methods

A systematic literature review was performed using the PubMed and Latin American and Caribbean Health Sciences Literature (LILACS) databases. Articles with original samples (cohorts and series reports) and systematic reviews published from January 1978 to June 2020 were included. The keywords used in the searches were “pediatric tracheostomy complications” and “children tracheostomy complications”. The search was limited to the pediatric age group (0–18 years old) and to articles written in English, Spanish and Portuguese.

The authors evaluated each of the titles and abstracts to insure that they met the inclusion criteria. Only articles with multiple series reports were selected, excluding case reports, non-systematic literature reviews, consensuses and guidelines. Articles that did not address the complications of the tracheostomy procedure were also eliminated, as well as those that were not found as full text or were duplicated.

The selection protocol followed the recommendations of the PRISMA (preferred reporting items for systematic reviews and meta-analyses) flow chart. Given the heterogeneity of the articles, it was not possible to perform a meta-analysis.

## Results

**Figure 1:**
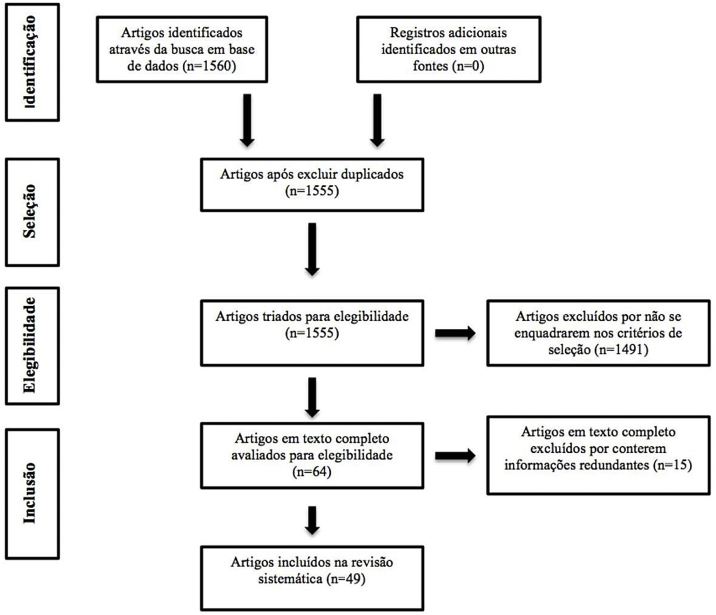
Literature review flowchart using the PRISMA strategy.

A total of 1560 articles were screened, based on the search parameters (Fig. 1). Of this total, 5 were duplicates and 1491 were also excluded in the screening process because they did not meet the inclusion criteria. Of the 64 full texts selected, it was decided to exclude 15 because they contained redundant information compared with other selected articles. Thus, we reached a total of 49 articles included in the systematic review, containing the most relevant and current information on the topic.

Table 1 shows the list of analyzed articles, with the total number of patients evaluated in each study and the rates of overall mortality and mortality directly related to the tracheostomy. With the exception of a prospective cohort,^3^ all the others are reports of case series or retrospective cohorts based on medical records reviews. In some articles it was possible to obtain the cause of death information for all patients. The time of follow-up evaluated in each study is heterogeneous, ranging from days to years, which limits the validity of the mortality rate analysis.

**Table 1:** Description of the articles regarding authorship, year of publication, sample size, mortality related to tracheostomy and overall mortality.

Author	Year of publication	n	Tracheostomy-related mortality (%)	Overall mortality (%)
Baker and Chorney^30^	2020	1607	–	–
Lee et al.^47^	2019	77	30 (38.9%)	30 (38.9%)
Syed et al.^18^	2019	50	0	9 (18%)
Maunsell et al.^29^	2018	160	2 (1.3%)	29 (18.1%)
Feehs et al.^41^	2018	57	–	–
Shay et al.^24^	2018	2248	–	118 (5.2%)
Schweiger et al.^17^	2017	123	–	38 (31%)
Dal'Astra et al.^1^	2017	5933	60 (1%)	734 (12.4%)
Ha et al.^40^	2017	164	–	–
Schwarz et al.^44^	2017	39	0	4 (10.3%)
Mahida et al.^6^	2016	206	12 (5.8%)	12 (5.8%)
Watters et al.^12^	2016	502	–	45 (9%)
Garcia-Urabayen et al.^27^	2016	25	1 (4%)	5 (20%)
McCaleb et al.^35^	2016	93	–	–
Levi et al.^46^	2016	264	0	58 (22%)
Douglas et al.^25^	2015	95	0	21 (19.9%)
Song et al.^26^	2015	111	2 (1.8%)	17 (15.3%)
Van Buren et al.^28^	2015	151	5 (3.3%)	19 (12.6%)
Olgivie et al.^31^	2014	248	5 (2%)	37 (15%)
Afolabi-Brown et al.^38^	2014	20	–	–
Provenzano et al.^48^	2014	9	–	–
Trey et al.^14^	2013	119	1 (0.8%)	25 (23%)
Cline et al.^37^	2012	170	–	–
Pérez-Ruiz et al.^3^	2012	249	8 (3.2%)	31 (12.5%)
Nobre et al.^21^	2011	27	0	4 (14.8%)
Genther and Thorne^16^	2010	421	–	–
Itamoto et al.^32^	2010	58	2 (3.4%)	–
Al-Samri et al.^33^	2010	72	1 (1.4%)	11 (15.3%)
Colman et al.^36^	2010	172	–	–
Tasca and Clarke^42^	2010	193	–	–
Özmen et al.^20^	2009	282	3 (1%)	34 (19%)
Pereira et al.^19^	2004	55	0	9 (16%)
Solares et al.^43^	2004	94	0	8 (8.5%)
Kremer et al.^9^	2002	25	0	5 (20%)
Greenberg et al.^10^	2001	200	–	–
Carr et al.^15^	2001	142	1 (0.7%)	21 (15%)
Nakanishi et al.^39^	2001	73	1 (1.4%)	–
Carron et al.^21^	2000	204	7 (3.6%)	39 (19%)
Park et al.^45^	1999	149	0	–
Coln et al.^4^	1998	76	–	–
Ward et al.^50^	1995	103	3 (2.9%)	37 (36%)
Citta-Pietrolungo et al.^5^	1993	30	–	–
Schlessel et al.^11^	1993	36	4 (11%)	13 (36.1%)
Rosenfeld and Stool^49^	1992	50	–	–
Gianoli et al.^8^	1990	60	1 (1.7%)	25 (42%)
Kenna et al.^7^	1987	124	3 (2.4%)	31 (25%)
Newlands and McKerrow^23^	1987	53	0	9 (17%)
Gaudet et al.^13^	1978	123	4 (3%)	18 (14.6%)
Perrotta and Schley^22^	1978	61	3 (4.9%)	–

(–) Represents insufficient information in the analyzed article.

Table 2 shows the total number of cases reported in the investigated literature according to each complication. Care was taken not to include in this group the number of articles analyzed in other systematic reviews that are also included in our study, to avoid duplication of data. Due to the heterogeneity of the studies, it is difficult to divide the total number of complications into early or late ones, since many studies did not bring this discrimination. Some of the complications are more accurately mentioned in some of the articles. As an example, some authors report granuloma according to its location, whereas others only use the term granuloma in a generic way. Thus, it was decided to group the reported complications under simplified terms. A considerable portion of the articles does not differentiate "total complications" from "total children with complications", since the same child may have developed more than one complication. Therefore, it is not possible to calculate these two values ​​precisely.

**Table 2:** Tracheostomy complications reported in the analyzed articles.

Complication	Total reported (%)
Skin lesions	1245 (23.7)
Granuloma	1073 (20.4)
Tracheocutaneous fistula	497 (9.5)
Accidental decannulation	438 (8.3)
Cannula obstruction	419 (8)
Skin infection	385 (7.3)
Pneumothorax	222 (4.2)
Pneumomediastinum	179 (3.4)
Tracheitis	148 (2.8)
Tracheal stenosis	138 (2.6)
Bleeding	94 (1.8)
Tracheo-innominate fistula	78 (1.5)
Pneumonia	61 (1.2)
Subglottic stenosis	58 (1.1)
Suprastomal collapse	51 (1)
Tracheomalacia	37 (0.7)
Subcutaneous emphysema	28 (0.5)
Air leak with impact on ventilation	23 (0.4)
Sepsis	15 (0.3)
Tracheal erosion	15 (0.3)
Glottic stenosis	13 (0.3)
False passage	10 (0.2)
Aspiration	7 (0.1)
Tracheoesophageal fistula	6 (0.1)
Suture dehiscence	3 (0.06)
Tracheal rupture	2 (0.04)
Stomal keloid	2 (0.04)
Stomal stenosis	1 (0.02)
Tracheal necrosis	1 (0.02)
Cricothyroid intubation	1 (0.02)

## Discussion

The incidence of tracheostomy complications ranges from 0% to 90% of the cases, with an average close to 40% in the numerous reported series.^4^, ^5^ There has been a downward trend over the past three decades.^1^ These complications vary in severity, ranging from intraoperatively controllable bleeding to death by decannulation or cannula obstruction.

There are some described risk factors associated with a higher frequency of complications, with age, birth weight and prematurity being some of them.^6^, ^7^, ^8^ The factors that can explain this higher occurrence in young children range from the need for prolonged tracheostomy use in this age group, through associated comorbidities, and finally the small diameter of the airway and of the cannula itself.^8^ There is scientific evidence showing that the incidence of complications associated with tracheostomy is higher in children than in adults.^9^ A tracheostomy performed in emergency situations significantly increases the risk of complications in relation to a controlled elective procedure; from 0.05% to 20%, with the main one being pneumothorax.^10^ Baseline disease and previous intubation (for mechanical ventilation) are also related to an increased risk.^7^ Patients with bronchopulmonary dysplasia, a comorbidity frequently associated with prematurity, have more abundant and viscous secretion, which increases the risk of obstructive complications.^11^ Presence of intraventricular hemorrhage and risky cardiac alterations also independently increase the chance of a major tracheostomy complication.^6^ Finally, in a retrospective multivariate cohort analysis of 502 tracheostomized children, those aged 1–4 years were 4 times more likely to have complications than those aged 13 or older; those with complex chronic conditions had a 3.3-fold greater risk to have complications than those without such conditions; and those with gastrostomy or peritoneal ventricle shunt, in addition to tracheostomy, had a 2-fold greater risk of complications than those without it (*p* < 0.01 for all comparisons).^12^

As for temporality, some authors consider early postoperative complications as the ones that occur within 72 h after the procedure, ^13^ while others consider a period within 7 days.^5^, ^7^ The Zurich classification was not used (perioperative complications are those occurring within 48 h of the tracheostomy and postoperative ones are those occurring after that), ^14^ as it does not discriminate between events occurring in the transoperative period from those occurring in the early postoperative period, making comparisons with other articles difficult. Table 3 shows a summary of the main complications, according to their temporality.

**Table 3:** Tracheostomy complications in children according to their temporality.

Transoperative
Hemorrhage, subcutaneous emphysema, pneumomediastinum, pneumothorax, cricoid incision, esophageal puncture, recurrent laryngeal nerve injury, incorrect cannula choice, selective intubation, cardiorespiratory arrest, death.

Postoperative
Early
Tracheostoma bleeding, accidental decannulation, cannula obstruction, local infection (cellulitis), peri-cannula air leak (difficulty in mechanical ventilation).
Late
Accidental decannulation, cannula obstruction, local and bronchial infection, suprastomal collapse, tracheal stenosis, tracheocutaneous fistula, tracheoesophageal fistula, tracheo-vascular fistula with innominate artery, subglottic stenosis, tracheal granuloma (distal and suprastomal), death.

### Transoperative complications

Complications that occur during surgery are usually related to the presence of interstitial air (pneumothorax, pneumomediastinum, subcutaneous emphysema). The reported incidence ranges from 0 or close to zero^1^, ^14^, ^15^, ^16^, ^17^, ^18^ to 28%.^9^ Of these, the most common one is pneumomediastinum. The most plausible explanation is the child's higher location of the pleural dome, which can sometimes reach the cervical region. Careful dissection technique, avoiding dissections performed laterally to the trachea, is the best way to avoid them. In small, asymptomatic pneumothorax and pneumomediastinum, the management is conservative. Pleural drainage may be necessary in larger ones. Subcutaneous emphysema can occur due to the closing of the edges of the postoperative wound with tight sutures or by making a very compressive dressing around the cannula. Neither measure is indicated. If it occurs, the opening of sutures and replacing the compressive dressing by a looser one are indicated.

The routine indication of chest radiography after tracheostomy has been discussed. In the literature, it seems that some services always require radiography.^10^, ^13^, ^19^ In asymptomatic and non-preterm patients, however, the current trend is that of non-indication,^16^ as there is a progressive decrease in the complications described in the previous paragraph.^14^, ^15^ Moreover, except when symptomatic, such complications do not require an intervention. It should be noted that cases of late pneumothorax, associated with the closure of tracheocutaneous fistula, have been described,^2^ in addition to those well known to be associated to mechanical ventilation with inadequate parameters.

Excessive bleeding during the surgical procedure may be due to injury to large cervical vessels or anomalous vessels, in addition to injuries to the thyroid gland and eventually the innominate artery. Again, the best prophylaxis is a careful dissection and the treatment is the ligation or cauterization of the vessel or bleeding point, respectively. Bleeding was ranked as the 3rd most frequent intraoperative complication in two studies, after pneumomediastinum and pneumothorax, in that order (in one study it occurred in 5% of children under 1 year and in 7% of older ones, and in the other, in 1.6% of cases).^9^, ^13^ A study reporting 37 years of experience in a Turkish service reported a 0.7% rate of intraoperative bleeding in 282 tracheostomies.^21^ A Brazilian study and an Indian study found no cases of transoperative bleeding.^17^, ^18^

Injury to recurrent nerves due to lateral dissections in the trachea, esophageal puncture due to poor cannula placement, cricoid incision in the case of very high tracheostomies and the false passage due to inadequate cannula insertion and positioning may all result from poor surgical technique and are not common. A North American series depicts this low frequency, finding an incidence of 0.8% of tracheoesophageal fistula.^13^ A Portuguese study reporting a 25 year experience with tracheostomy evaluated 27 patients and found a case of transoperative tracheoesophageal fistula (3.7%).^21^ The false passage is not so uncommon and can occur in 7% of preterm babies^22^ and in 0.8%^13^ to 16% of older children.^23^ It can also occur in the postoperative period, after accidental decannulation and an inaccurate attempt to reposition the cannula, usually by family members and even health professionals who are not appropriately trained. It is a frequent cause of return to the hospital after discharge.^24^

Selective intubation usually occurs in preterm infants or even older children in the case of lower neck tracheostomies, where cannulas with adequate diameters, but very long, are chosen.

Finally, cardiorespiratory arrest during tracheostomy is extremely rare, described at a frequency of 0.8%.^13^

### Early postoperative complications

Bleeding in the immediate postoperative period of the stoma construction has a relatively low prevalence, ranging from 1.8%^19^ to 5.2% of children.^25^ In general, it is the result of inadequate hemostasis during the procedure and can be significant for a neonate. Surgical revision may be necessary.^19^

Accidental primary decannulation is a severe complication and its incidence ranges from 0.8%^13^ and 0.9%^26^ to 20%^15^, ^27^ in the literature. In the series of 25 tracheostomized patients in Barcelona, ​​20% had accidental decannulation, all in the first 48 h (80% in the first 24 h). Cardiorespiratory arrest occurred in 80% of patients who underwent decannulation, with death occurring in one.^27^ In a Scottish series, cannula displacement occurred in 5.2% of cases in the early postoperative period.^25^ A Brazilian series showed an accidental decannulation rate of 2.44%.^17^ Another series, reporting 30 years of experience with pediatric tracheostomy at a university hospital in Zurich, found a 13% cannula displacement rate that occurred 48 h after the procedure, but without discriminating at what moment it occurred, making it difficult to classify it as an early or late postoperative occurrence.^14^ As the consequences of decannulation can be dramatic, a good practice is to use containment sutures on the anterior tracheal wall that exit the stoma or, according to the most current trend, to mature the stoma,^18^ especially in neonates and small infants, facilitating the quick and correct repositioning of the cannula in case of displacement. Another practice, less often used, is to make one or two anchoring stitches from the plastic cannula to the skin.

While cannula obstruction by a blood clot is usually an early postoperative complication, the one caused by a mucus plug is generally a later one. However, in cases with underlying disease with alterations in the quantity and quality of the secretion, especially in preterm infants, the plug may also occur early. Early cannula obstruction occurred in 4% of tracheostomized patients in a pediatric ICU^27^ and in 4.6% of patients up to the 4th postoperative day in a study that sought to evaluate the safety of cannula replacement on the 3rd day after tracheostomy, with no fatal cases.^28^ A Korean article, describing a tracheostomy technique with a vertical tracheal incision and stoma maturation, reported a cannula obstruction rate of 0.9%, with none leading to the patient's death,^26^ as in an Indian study that also used the stoma maturation technique, but using a circular tracheal incision.^18^ The continuous administration of humidified air until the first cannula change can reduce this type of complication. Another cause of cannula obstruction, the false passage, has been described in 3.6% of cases in a Brazilian sample.^17^

The local infection (cellulitis), which occurs in the earliest stages, manifests as hyperemia of the peristomal skin, and hardening and increased local heat may occur. There may be greater or lesser amounts of secretion. Infection was found in 1.6%–14.6% of tracheostomized children.^17^, ^19^, ^28^, ^29^ Treatment includes cannula replacement - traditionally recommended up to a maximum of 5–7 days, and can be safely removed on the 3rd day,^28^ local hygiene, use of antibiotic ointments and, sometimes, oral antibiotic therapy with spectrum for the most common respiratory microorganisms. Cautery overuse in the dissection of subcutaneous tissue and infrahyoid muscles, with excessive tissue destruction, is listed as one of the potential causes.^19^ The appearance of skin lesions such as irritation of the peri-tracheostoma skin and pressure ulcers can be minimized with the use of waterproof dressings with or without antimicrobial properties, as well as the use of Velcro fasteners instead of cotton laces, since the first ones tend to accumulate less moisture and secretion.^30^

Excessive air leakage around the tracheostomy cannula is another early complication of tracheostomy and can be a problem in a tracheostomized patient who requires respiratory support with high ventilatory parameters. It can occur in up to 14.6% of preterm newborns, especially those with very low birth weight (< 1 kg).^19^ It can also occur in infants, as demonstrated by Carr et al. in their series, with an incidence of 7% of patients before the first cannula change.^15^

### Late postoperative complications

Decannulation was the most frequent late complication of tracheostomy (6%) in a Canadian cohort with 30 years of follow-up. It was the cause of death in 2% of these children.^31^ A similar incidence of late decannulation was found in a Korean study (2.7%),^26^ which resulted in the death of one patient at home. In a Brazilian series evaluating 58 children, decannulation occurred in 3 (5.7%) patients, one of which had cardiopulmonary arrest that was reversed, whereas the other died.^32^ In another Brazilian series, the incidence was the same, occurring in 5.7%, with no fatal cases.^17^ An Indian study found no cases of accidental decannulation.^18^

Obstruction by a mucus plug occurred in 10% of patients in a study carried out in Calgary, Canada.^33^ All had cardiorespiratory arrest and there was one death. All obstructions occurred between 7 and 90 days after the tracheostomy. A lower incidence was observed in a Brazilian series (4.1% of patients),^17^ and in a Korean series (only 1 case — 0.9%).^26^ Not only the mucous plug, but also granulomas and false passage can lead to cannula obstruction. This complication can be prevented with correct care of the tracheostomy cannula since the first postoperative days, involving aspirations and periodic cannula changes. Frequent aspirations, and the instillation of saline solution to help in the humidification of mucus can also be performed, are essential to avoid obstruction by dry secretion in the cannula lumen.^34^

Local and low respiratory infections were the most frequent complications in some studies,^15^ affecting up to 90% of patients.^33^ Perhaps part of this increased incidence of infection is due to the non-differentiation between colonization and infection. Long-term tracheostomized children may become colonized by *S. aureus*, including those resistant to methicillin, and / or *P. aeruginosa* and other Gram-negative microorganisms.^35^ It is necessary to differentiate between these two conditions. Colonization does not require treatment, unless there is a sign of acute infection or before reconstructive airway procedures. Surgical wound infection can occur in any surgical procedure and should be treated with a short course of oral antibiotics. The International Pediatric Otolaryngology Group in its 2016 consensus recommends the use of routine antimicrobial prophylaxis at least until the first cannula change. Tracheitis can be an early or late complication. It occurred late in 48.8% of 156 cases.^36^ In the case of tracheitis, routine cultures of the prosthesis or tracheal secretions is not recommended.^37^ In cases of lower airway infections (bacterial bronchitis and pneumonia), the method of choice for collecting secretions for bacterial culture is the bronchoalveolar lavage, preferably the “protected” type, carried out by a catheter that goes through the biopsy channel of the bronchoscope, preventing contamination by secretions from the upper airways.^38^

Suprastomal collapse, subglottic stenosis and tracheal stenosis have similar mechanisms, resulting from damage to the mucosa and tracheal cartilage, and can progress with partial absorption of cartilage, fibrosis and scar stenosis. The suprastomal collapse results from the weakening of the anterior tracheal wall, which is generally superior to the tracheostoma, and can be prevented by fixing the tracheal wall to the tracheostoma skin at the time of the surgery. Its occurrence varied between 0.2% and 13.1% in two Brazilian studies.^1^, ^29^ Subglottic stenosis is usually secondary to an inadvertent injury to the cricoid cartilage during the procedure or injuries induced by previous intubation. A Brazilian study reported the highest incidence of subglottic stenosis found in the literature, 27.4%, associated with tracheostomy.^39^ Tracheal stenosis, on the other hand, can occur above, at the site or below the tracheostoma, as far as the tip of the cannula reaches the tracheal wall, including the carina region. It can also be secondary to tracheal injuries resulting from previous intubation or even the repeated aspirations with catheters performed using an inadequate technique. The incidence of tracheal stenosis after tracheostomy ranged from 0.4%^3^ to 12% of cases.^9^

The tracheocutaneous fistula (TCF), that is, the epithelialization of the trajectory from the skin to the trachea (mature stoma), is desirable in the early postoperative period, but it can become a long-term problem, preventing the spontaneous closure of the tracheostoma. Most tracheostomas close spontaneously through healing by secondary intention. The incidence of TCF in the series varies between 3.1% and 57.3%.^1^, ^40^, ^41^ A study that reviewed the 14-year experience of pediatric tracheostomy in a hospital in Liverpool showed a percentage of 11.9% of need for surgical closure of the TCF. These were children with long-term tracheostomies (at least two years, with a median of 4 years) who had undergone the surgical procedure before 1 year of age.^42^ This hypothesis was also corroborated by a North-American study, which demonstrated a significantly increased relative risk of developing tracheocutaneous fistula in patients who remained tracheostomized for more than 24 months.^42^ Currently, there is a tendency among surgeons to mature the stoma early. There is a concern that this surgical approach will increase the TCF rate, mainly because with the use of some techniques in which maturation was universal, such as “starplasty”, this occurs in practically 100% of cases.^43^ However, this assertion has not been confirmed in an Israeli service experience.^44^ Moreover, one of the first comparative studies showed similar incidences between matured and unmatured stomas, 10.2% and 12.8%, respectively. Also, there was not a higher incidence of granulation tissue formation in the mature stoma.^45^ Another study that compared the overall complications of tracheostomy between matured and non-matured stomas also found no statistically significant differences, including TCF.^36^ More recently, using a simple technique without the construction of flaps, with only two sutures in the inferior-lateral region of the stoma joining the subcutaneous tissue with the pretracheal fascia, the TCF rate in matured and non-matured stomas was similar (27% vs. 22%, respectively).^46^

A tracheo-vascular fistula with the innominate artery is a rare complication,^2^, ^15^ described in articles that show successful surgical repair techniques. The innominate artery injury that can occur during the transoperative period in very low tracheostomies (below the 4th tracheal ring) is even rarer and is caused by direct trauma to the artery, especially in cases of arteries located at a higher position in the neck. This bleeding should not be mistaken for recurrent bleeding, of different intensities, where the tracheostomy cannula can cause local irritation and late hemorrhage and inflammation, with bleeding secondary to the presence of granulomas, especially in the case of tracheal aspirations performed with less delicate care. The true innominate artery fistula is usually a late event (many cases are reported in the 3rd week after the tracheostomy) and may occur when the tip of a poorly positioned tracheostomy cannula (mainly metallic ones) causes friction and results in erosion and ulceration on the anterior tracheal wall and on the posterior wall of the innominate artery. Precipitating factors comprise the use of cannulas that have cuffs with excessive pressure, low tracheal incision and hyperextension of the neck. Its incidence is low, less than 1%, as demonstrated in a large Spanish multicenter cohort, in which there was only one case (0.4%).^3^ It must be recognized quickly as it requires emergency surgical intervention. In some cases, more temporary “sentinel” bleeds can indicate a devastating hemorrhage. A clinical team trained to stabilize the patient for emergency surgery is important in the acute phase. When unrecognized, inadequately stabilized prior to surgery and untreated surgically as an emergency case, the innominate artery fistula is associated with death in practically 100% of affected cases. However, when adequately managed in a timely manner, it can have a more favorable outcome, as demonstrated by a recent systematic review that found 38.6% of mortality in the described cases.^47^

The late tracheoesophageal fistula is a rare occurrence, with an incidence of less than 1%. It occurs due to the necrosis of the posterior tracheal wall, usually secondary to the use of poorly positioned cannulas with cuffs or metallic ones. There are more reports in the literature mentioning it as a tracheostomy complication in adults. In a study that analyzed the efficacy of sliding tracheoplasty in the repair of tracheoesophageal fistulas, one of 9 cases of operated fistulas was secondary to complications caused by the tracheostomy cannula.^48^

It is a debatable topic in the literature whether tracheal granulation or granuloma represents an actual complication of tracheostomy, as it is often asymptomatic.^1^ It has an estimated frequency of 12.3% to 66% of cases.^33^ It was the main late complication of tracheostomy in at least two studies, published in 1993 and 2018.^5^, ^29^ The recent series that studied ventilator-dependent institutionalized cases showed the presence of peristomal granulomas in 40.6% and suprastomal in 37.5% of children. Taking into account the low incidence of large and obstructive granulomas and the failure of the surgical excision to decrease their recurrence, it was concluded that excision was not recommended in children with non-obstructive granulomas with stable tracheostomies.^49^ However, when considering decannulating the patient, a previous endoscopy and excision of granulomas that may hinder the process is mandatory. Management can be endoscopic, using different instruments, or transtracheostoma. In the case of granulomas that are too large or firm (mature) to be removed through endoscopic techniques, or too large or firm to be removed via the stoma, open excision is indicated.

Death is a potential undesirable event in tracheostomized patients. Most of the time, it is not correlated with the procedure itself, but occurs secondary to the evolution of the underlying disease. The overall mortality rate varies between case series, and reach up to 40%, while mortality from the procedure itself can reach up to 6% in the pediatric population.^7^, ^9^, ^13^ Children with heart and neurological problems have a higher overall mortality rate when compared to children with craniofacial malformations or upper respiratory obstruction.^9^ Preterm children and extremely low-birth-weight infants also have higher mortality rates.^50^ A recent study showed that children under 1 year of age have a seven-fold higher risk of death when compared to children over 1 year.^12^ Morbidity and mortality rates significantly depend on how well trained the medical staff is and how well informed are the patients’ parents and caregivers, since the main causes of death are accidental decannulation and stoma blockage by secretion plug.^1^ Low rates of complications and death are also correlated to the elective procedure, in referral hospitals and by experienced, well-trained surgeons.^19^

## Conclusion

Childhood tracheostomy is a relatively common procedure in tertiary hospitals for a variety of reasons – from neurological sequelae to cases of mechanical airway obstruction. Regardless of the reason and technique used, several complications have been described and their prevalence rates vary widely in the literature. Among the most frequent complications are granulomas, skin infections, cannula obstruction and persistence of the tracheocutaneous fistula in patients who can be decannulated. Moreover, the mortality rate from causes exclusively related to the tracheostomy is low, but the overall mortality of tracheostomy patients is considerable, and increases according to the associated comorbidities, emergency procedure, young age and low weight. Adequate training of the assistance team and support and training of parents regarding hygiene procedures and eventual repositioning of the cannula before discharge can help minimize these events.

## Conflicts of interest

The authors declare no conflicts of interest.
